# An Optimized PCR Assay to Detect *Escherichia Coli* Harboring the *astA* Gene Encoding the Enteroaggregative *E. coli* Heat-Stable Enterotoxin 1 in Various Food Matrices

**DOI:** 10.14252/foodsafetyfscj.D-25-00013

**Published:** 2025-12-19

**Authors:** Sakura Arai, Nobuyo Ikeda, Mayumi Kadoguchi, Emi Arikawa, Akito Mizokoshi, Kaori Shimmen, Koji Yokoyama, Rie Doi, Dai Saiki, Jun Yatsuyanagi, Shouhei Hirose, Takahiro Ohnishi, Yukiko Hara-Kudo

**Affiliations:** 1 Division of Microbiology, National Institute of Health Sciences, 3-25-26, Tonomachi, Kawasaki-ku, Kawasaki, Kanagawa 210-9501, Japan; 2 Division of Biological Science, Hiroshima City Institute of Public Health, 4-1-2, Shoukousenta, Nishi-ku, Hiroshima 733-8650, Japan; 3 Microbiology Division, Kumamoto Environmental Research Center, 404-1, Ezumachitokorojima, Higashi-ku, Kumamoto 862-0946, Japan; 4 Division of Microbiology, Kitakyushu City Institute of Health and Environmental Sciences, 1-2-1 Shinike, Tobata-ku, Kitakyushu, Fukuoka 804-0082, Japan; 5 Oita Prefectural Institute of Health and Environment, 2-8, Takaenishi, Oita 870‒1117, Japan; 6 Himeji City Institute of Environment and Health, 3 Sakatamachi, Himeji, Hyogo 670-8530, Japan; 7 Fukui Prefectural Institute of Public Health and Environmental Science, 39-4, Harame-cho, Fukui-shi, Fukui 910-8551, Japan; 8 Saitama Institute of Public Health, 410-1, Ewai, Yoshimi-machi, Hiki-gun, Saitama 355-0133, Japan; 9 Tokyo Metropolitan Institute of Public Health, 3-24-1, Hyakunin-cho, Shinjuku, Tokyo 169-0073, Japan; 10 Yuri-Honjyo Public Health Center, 408 Mizubayashi, Yuri-Honjyo City, Akita 015-0885, Japan; 11 Department of Microbiology, Hoshi University, 2-4-41 Ebara, Shinagawa-ku, Tokyo 142-8501, Japan

**Keywords:** *astA*, *Escherichia coli*, food, PCR, detection

## Abstract

Diarrheagenic *Escherichia coli* strains harboring the *astA* gene which encodes for the enteroaggregative *E. coli* heat-stable enterotoxin 1 (EAST1) have been implicated in several foodborne outbreaks in Japan even in the absence of any other specific virulence marker of each pathovar. The polymerase chain reaction (PCR) assay is a critical tool used for detection and many *astA* specific primer sets have been developed though their specificity and sensitivity for *astA* detection directly from food samples have not been evaluated. Herein, four distinct PCR primer sets and three enzymes were evaluated in enriched food cultures to optimize *astA* detection. The Yamamoto & Echeverria PCR method yielded clear, easily interpretable results with high intensity of PCR product or no products. Combining this primer set with the Quick Taq HS DyeMix enzyme resulted in *astA-*specific amplicons without non-specific products from food cultures, indicating the superiority of this system in detecting *astA* in food samples. Furthermore, this primer set demonstrated the highest consistency with the *E. coli* harboring *astA*-isolation results. Subsequently, this system exhibited high specificity and sensitivity with a ≤5 log CFU/mL detection limit. These findings suggest that combining the Yamamoto & Echeverria primer set and the Quick Taq HS DyeMix offers an effective tool for detecting *astA* in food samples. We anticipate this PCR assay will enhance the detection and subsequent isolation of *E. coli* strains harboring *astA* from food products.

## 1. Introduction

Enteroaggregative *Escherichia coli* (EAEC) is a bacterium associated with persistent diarrhea particularly in young children. Some EAEC strains produce a low-molecular-weight heat-stable enterotoxin designated EAEC heat-stable enterotoxin 1 (EAST1)^1^^)^. This toxin is a 38-amino acid peptide with a molecular weight of 4,100 Da that exhibits 50% sequence similarity with the enterotoxic domain of the heat-stable enterotoxin a (STa) produced by enterotoxigenic *E. coli* (ETEC)^2^^)^. The gene encoding EAST1 (*astA*) is a 117-base pair (bp) DNA sequence, and its prevalence in EAEC originated from human diarrheal stool ranges from 26 to 87%^3^^,^^4^^)^.

In Japan, some diarrheagenic *E. coli* isolated from foodborne infection outbreaks and sporadic diarrhea cases lacked any other specific virulence marker of each pathovar other than *astA*^5^^,^^6^^,^^7^^,^^8^^,^^9^^,^^10^^,^^11^^)^. In a large-scale foodborne outbreak in June 2020, 2,958 patients were reported, and *E. coli* serotype O7:H4 harboring *astA* was isolated from both patients and red seaweed (*Gigartina tenella*) used in seaweed salad^12^^)^ of school lunches. However, the food associated with most other outbreaks remain unidentified. Therefore, a specific and sensitive method for detecting *astA*-harboring *E. coli* from the food of foodborne outbreaks is essential.

Polymerase chain reaction (PCR) assays are valuable tools for detecting *astA* in various samples. Many conventional^3^^,^^13^^,^^14^^,^^15^^,^^16^^,^^17^^)^ and real-time PCR^18^^)^ assays targeting *astA* have been developed. However, the applicability of these PCR assays in complex food matrices, which can contain PCR inhibitors, remains unevaluated. Therefore, in this study, PCR assays were performed to identify the most effective method for detecting *E. coli* strains harboring *astA* in food samples, aiming to improve food safety and outbreak investigations.

## 2. Materials and Methods

### 2.1 Bacterial Strains

Thirty-nine bacterial strains were used in this study, including nine *E. coli* strains harboring *astA* (AST19, AST20, AST46, AST73, AST81, AST85, AST198, AST204, and AST205) isolated from foodborne outbreaks (**Table S1**). *Vibrio parahaemolyticus* and *Morganella morganii* subsp. *morganii* were cultured in tryptone soy broth (TSB; Oxoid, Hampshire, United Kingdom) supplemented with 2% NaCl. All other strains were cultured in TSB. All strains were incubated at 37°C for 18 h.

### 2.2 Food Samples and Enrichment

Nineteen food samples were purchased from a supermarket in Japan between January and May 2022. These samples included three baby corns (Nos. 1–3), one beef omasum tripe, one beef large intestine, two chicken thighs (Nos. 1–2), one mackerel, five okras (Nos. 1–5), one sliced pork, two pork livers (Nos. 1–2), one sea bass, and two white-leg shrimps (Nos. 1–2). All the samples were cut into small pieces and thoroughly mixed prior to use.

For one baby corn (No. 2) and two okra (Nos. 4–5), three separate stomacher bags (n = 3), each containing 25 g of the respective sample were prepared. These samples were homogenized in 225 mL of modified *E. coli* broth (mEC) supplemented with novobiocin (NmEC; Eiken Chemicals, Tokyo, Japan) for 1 min. For the remaining 16 food samples, 25 g of each was placed in a single stomacher bag (n = 1) and homogenized in 225 mL of mEC (Nissui, Tokyo, Japan) for 1 min. All samples were incubated under static conditions at 42°C for 22 h.

### 2.3 DNA Extraction

DNA from the *E. coli* strains harboring *astA*, used as a positive control for the PCR assay, was extracted using the boiling method described previously^19^^)^. DNA of the colonies grown on agar plates was also extracted using the boiling method. Genomic DNA from all bacterial strains was extracted using a NucleoSpin Tissue kit (Machrey-Nagel, Düren, Germany) according to the manufacturer’s instructions. This DNA was used for the specificity assay of the PCR method detailed below. DNA from enriched food cultures was extracted using the alkaline DNA extraction as described previously^20^^)^.

### 2.4 PCR Method

Four conventional PCR primer sets, as reported by Ito et al^13^^)^, Hidaka et al^18^^)^, Müller et al^15^^)^, and Yamamoto & Echeverria^3^^)^ were used in the present study (**Table 1**). Although the PCR assay reported by Hidaka et al^18^^)^ was originally designed as a real-time PCR assay using a probe, only the forward and reverse primers were used in this study. The PCR assay reported by Müller et al^15^^)^ was originally designed to detect 12 genes (*escV, bfpB, stx1, stx2, elt, est1a, est1b, invE, astA, aggR, pic,* and *uidA*); however, only the PCR primer set specific for *astA* was used for evaluation in this study. Genomic DNA extracted from *E. coli* strains harboring *astA* were used as a positive control.

**Table 1.** PCR primers used in this study

Primer name	Sequence (5’-3’)	Product size (bp)	Reference (No.)
EASTOSl	GCCATCAACACAGTATATCCG	109	Ito et al, 2014 (13)
EASTOAS2	CGCGAGTGACGGCTTTGTAG		
astA-61f	ATGCCATCAACACAGTATATCCG	111	Hidaka et al, 2009 (18)
astA-171r	CGCGAGTGACGGCTTTGTA		
MP-astA-F	TGCCATCAACACAGTATATCCG	102	Müller et al, 2007 (15)
MP2-astA-R	ACGGCTTTGTAGTCCTTCCAT		
EAST11-a	CCATCAACACAGTATATCCGA	111	Yamamoto & Echeverria, 1996 (3)
EAST11-b	GGTCGCGAGTGACGGCTTTGT		

The PCR amplification using the AmpliTaq Gold DNA polymerase (Thermo Fisher Scientific, Waltham, MA) was carried under the following conditions: 10 min at 95°C, 30 cycles of 30 s at 95°C, 30 s at 55°C, and 1 min at 72°C followed by final extension for 2 min at 72°C. PCR amplification using the Quick Taq HS DyeMix (Toyobo, Osaka, Japan) was conducted under the following conditions: 2 min at 94°C, 30 cycles of 30 s at 94°C, 30 s at 55°C, and 1 min at 68°C, followed by final extension for 1 min at 72°C. PCR amplification using the Ex Taq DNA polymerase (TaKaRa Bio, Shiga, Japan) was applied under the following conditions: 10 s at 98°C, 30 cycles of 10 s at 98°C, 30 s at 55°C, and 1 min at 72°C, followed by final extension for 1 min at 72°C. The resulting PCR products were electrophoresed on a 2% (w/v) agarose gel in Tris-borate-EDTA (TBE) buffer at 100 V for 45 min. The gels were stained with GelRed (Biotium, Hayward, CA, USA), and the bands were visualized and imaged under ultraviolet (UV) illumination. The sizes of the PCR amplicons were determined by comparison to a 100-bp DNA ladder (EZ Load Molecular Ruler, Bio-Rad, Hercules, CA, USA).

### 2.5 Isolation of *E. coli* strains Harboring *astA* from Enriched MEC Cultures

For the 16 mEC-enriched food cultures, the extracted DNA was subjected to PCR amplification using the primer set described by Müller et al^15^^)^ and the Quick Taq HS DyeMix. Enriched cultures that showed *astA*-specific PCR band were subsequently streaked onto CHROMagar ECC (CHROMagar Microbiology, Paris, France) and incubated at 37°C for 24 h. Twenty presumptive *E. coli* colonies, as suggested by the blue coloration, were selected and DNA was extracted from each. PCR assay using the primer set by Müller et al^15^^)^ was then performed to confirm the identity of these isolates as *E. coli* strains harboring *astA*. The PCR conditions were identical to those described above.

### 2.6 Evaluation of PCR Primer Sets for *astA* detection in Food Samples

Food samples prepared individually in three stomacher bags (n = 3) were used as follows: DNA extracted from the NmEC-enriched cultures of baby corn No. 2 (stomacher bag Nos. 1–3), and okra Nos. 4 and 5 (stomacher bag Nos. 1–3). Four kinds of PCR assays described by Ito et al^13^^)^, Hidaka et al^18^^)^, Müller et al^15^^)^, and Yamamoto & Echeverria^3^^)^ were performed. The PCR amplification was carried out with the TaKaRa Ex Taq DNA polymerase (TaKaRa Bio), and the reaction mixtures were prepared according to the manufacturer’s instructions. The thermal cycling conditions were identical to those described above.

### 2.7 Evaluation of PCR Enzymes for *astA* detection

Enriched cultures of okra No. 4 in stomacher bags 1 and 3 were spread on tryptic soy agar (TSA; BD Biosciences, Franklin Lakes, NJ, USA). The enriched culture of okra No. 4 in stomacher bag No. 1 was also spread onto CHROMagar STEC (CHSTEC; CHROMagar, Paris, France). The agar plates were then incubated at 37°C for 22 h. Colonies grown on these agar plates were harvested and their DNA was extracted. A PCR assay using the primer set described by Yamamoto & Echeverria^3^^)^ was then performed using the following three PCR DNA polymerases: AmpliTaq Gold, Quick Taq HS DyeMix, and TaKaRa Ex Taq. The PCR conditions were identical to those described above.

### 2.8 Evaluation of PCR Primer Sets with Quick Taq HS DyeMix for Detection in Enriched Food Cultures

Among the 16 mEC-enriched food cultures, those that showed *astA*-specific PCR amplicon were selected for further analysis. These samples were categorized into two groups: group 1, from which no *E. coli* strain harboring *astA* was isolated from the 20 colonies tested, and group 2, from which at least one *E. coli* strain harboring *astA* was isolated from the 20 colonies tested. The DNA extracted from each of these enriched cultures was then used as a template for PCR assays employing the primer sets described by Ito et al^13^^)^, Müller et al^15^^)^, and Yamamoto & Echeverria^3^^)^ with Quick Taq HS DyeMix as the DNA polymerase. Thermal cycling conditions were identical to those described above.

### 2.9 Specificity and Sensitivity Evaluation of the PCR Assay

The specificity of the PCR primer set described by Yamamoto & Echeverria^3^^)^ in conjunction with Quick Taq HS DyeMix was assessed using the genomic DNA of the bacteria species listed in Table S1. The sensitivity of this PCR primer set was evaluated using four *E. coli* strains harboring *astA*: AST19, AST46, AST73, and AST205, as described previously^21^^)^. Briefly, serial 10-fold dilutions (10^−1^ to 10^−6^) of each bacterial culture were prepared in conjunction with the enriched cultures of the baby corn No. 3, sliced pork, and white-leg shrimp No. 2. DNA was extracted and used as the template for PCR amplification. The highest bacterial dilution that yielded a positive PCR result in triplicate was defined as the detection limit.

To quantify the initial bacterial inoculum, a 100 μL aliquot of the appropriate bacterial dilution was plated onto TSA. Colonies were counted after incubation at 37°C for 18 h.

## 3 Results

### 3.1 Isolation of *E. coli* strains Harboring *astA* from Enriched Food Cultures

Among the 16 enriched food cultures examined, *astA*-specific PCR amplicons were detected by agarose gel electrophoresis in 13 cultures. Subsequently, *E. coli* strains harboring *astA* were isolated from five of these PCR-positive cultures (**Table 2**).

**Table 2.** Isolation of *E. coli* strain harboring *astA* from enriched food cultures and intensities of *astA*-specific PCR product of three PCR primer sets

Group	Isolation of *E. coli* strain harboring *astA* from food	Food	PCR primer set		
			Ito et al	Müller et al	Yamamoto & Echeverria
Group 1	-	Chicken thigh No. 1	+	+	+
		Pork liver No. 1	±	+	-
		Beef large intestine	±	+	+
		Whiteleg shrimp	+	+	+
		Mackerel	+	+	+
		Okra No. 1	+	+	-
		Okra No. 2	+	+	+
		Baby corn No. 1	+	+	+
Group 2	+	Chicken thigh No. 2	+	+	+
		Pork liver No. 2	+	+	+
		Beef omasum tripe	+	+	+
		Sea bass	+	+	+
		Okra No. 3	+	+	+
		Positive control	+	+	+

DNA extracted from enriched culture of food was tested by PCR with PCR enzyme of Quick Taq HS DyeMix.

+ showed specific PCR product.

± showed specific but faint PCR product.

- showed no PCR product.

### 3.2 Evaluation of PCR Primer Sets for *astA* detection in Enriched Food Cultures

The results obtained with the four PCR primer sets were consistent across the enriched cultures from stomacher bag Nos. 1 and 2 of baby corn No. 2, and in stomacher bag No. 3 of okra No. 4 (**Table 3**). However, for all other stomacher bags, the intensity of the *astA*-specific PCR bands varied among the four PCR primer sets. The *astA*-specific PCR amplicon was not detected with the primer set described by Yamamoto & Echeverria in these other samples.

**Table 3.** Comparison of the intensity of PCR product of four PCR primer sets

Food	Stomacher bag No.	PCR primer set			
		Ito et al	Hidaka et al	Müller et al	Yamamoto & Echeverria
Baby corn No. 2	No. 1	+	+	+	+
	No. 2	+	+	+	+
	No. 3	+	+	+	-
Okra No. 4	No. 1	±	-	-	-
	No. 2	±	-	±	-
	No. 3	-	-	-	-
Okra No. 5	No. 1	+	+	±	-
	No. 2	+	-	-	-
	No. 3	+	+	+	-
Positive control		+	+	+	+

+ showed specific PCR product.

± showed specific but faint product.

- showed no PCR product.

### 3.3 Evaluation of PCR Enzymes for *astA* detection in Food Cultures

PCR amplifications conducted with AmpliTaq Gold as the DNA polymerase did not yield any specific or nonspecific amplicons from the DNA of the tested food cultures (**Table 4**). Moreover, the intensity of the *astA*-specific PCR amplicon from the positive control was weaker than that observed with the other two enzymes. The TaKaRa Ex Taq DNA polymerase also did not yield any *astA*-specific bands from food culture DNA samples; however, it generated multiple non-specific bands. The intensity of the *astA*-specific PCR amplicon of the positive control was comparable between the reactions that used the TaKaRa Ex Taq and Quick Taq HS DyeMix. Conversely, the PCR amplifications using the Quick Taq HS DyeMix DNA polymerase did not yield any specific or nonspecific bands in the DNA analysis from the food cultures.

**Table 4.** Optimization of PCR assay with the Yamamoto & Echeverria primer using three different PCR mixes

Food	Stomacher bag No.	Agar plate	PCR enzyme							
			AmpliTaq Gold			Quick Taq HS DyeMix			TaKaRa Ex Taq	
			Specific band	Non-specific band		Specific band	Non-specific band		Specific band	Non-specific band
Okra No. 4	No. 1	TSA	-	-		-	-		-	+
	No. 1	CHSTEC	-	-		-	-		-	+
	No. 3	TSA	-	-		-	-		-	+
Positive control			+*	-		+	-		+	+

DNA used in PCR assay was extracted from the colonies swept from the respective agar plates.

TSA: tryptic soy agar; CHSTEC: CHROMagar STEC.

+ showed specific PCR product.

- showed no PCR product.

* The intensity of the *astA*-specific PCR amplicon from the positive control was weaker than that observed with the other two enzymes.

### 3.4 Evaluation of PCR Primer Sets with Quick Taq HS DyeMix for *astA* detection in Enriched Food Cultures

Among the food samples from group 1, where *E. coli* strains harboring *astA* were not isolated, all three PCR primer sets yielded *astA*-specific PCR amplicons in six samples-(**Table 2**). Pork liver No. 1 and okra No.1 yielded *astA*-specific PCR amplicons with the primer sets described by Ito et al. and Müller et al. However, no *astA*-specific PCR amplicon was detected with the primer set described by Yamamoto & Echeverria in these two samples. Conversely, all five tested samples from group 2, where *E. coli* strains harboring *astA* were isolated, yielded *astA*-specific PCR amplicons for all three PCR primer sets.

### 3.5 Specificity and Sensitivity of the Optimized PCR Assay

The combination of PCR primer set described by Yamamoto & Echeverria and Quick Taq HS DyeMix specifically amplified the *astA* gene from all nine *E. coli* strains harboring *astA* in this study (Supplementary **Table S1**). All the other 30 bacterial strains analyzed in this study yielded negative results in this assay.

Among the three kinds of food matrices analyzed in this study, the detection limits of the PCR assay using the primer set described by Yamamoto & Echeverria were 4.0 log CFU/mL or lower for *E. coli* strains AST19, AST46, and AST73, and was 5.0–5.1 log CFU/mL for strain AST205 (Table 5). When the detection limits were assessed in enriched cultures of baby corn, sliced pork, and white-leg shrimp spiked with the four *E. coli* strains, the ranges were 3.8–5.0 log CFU/mL, 3.0–5.1 log CFU/mL, and 3.0–5.1 log CFU/mL, respectively.

**Table 5.** Detection limits of the optimized *astA*-specific PCR assay with enriched food cultures

*E. coli* strain harboring *astA*	Food		
	Baby corn No. 3	Sliced pork	White-leg shrimp No. 2
AST19	3.9*	3.0	3.0
AST46	3.8	3.1	3.1
AST73	4.0	3.5	3.5
AST205	5.0	5.1	5.1

PCR assay by Yamamoto & Echeverria was performed with Quick Taq HS DyeMix.

* Detection limit (log CFU/mL) is shown.

## 4 Discussion

To identify the causative agents of foodborne outbreaks, it is crucial to detect and isolate foodborne pathogens accurately. Since the *astA* gene is only a 117-bp-long DNA sequence, PCR primers specific for *astA* were designed at almost the same region within the 5′- and 3′-ends of the gene^3^^,^^13^^,^^14^^,^^15^^,^^16^^,^^17^^,^^18^^)^. Notably, in a survey of foodborne disease outbreaks that occurred in 2019^22^^)^, bacterial strains positive for the *astA*-specific PCR assay developed by Yatsuyanagi et al^17^^)^ were isolated from food samples. However, these strains were PCR-negative with primers described by Ito et al^13^^)^ (data not shown). Furthermore, false-positive PCR amplicons originating from *Enterobacter* spp. were seen in the *astA*-specific PCR assay developed by Yatsuyanagi et al^17^^)^ (data not shown). Accordingly, four distinct primer sets were selected and their ability to detect *astA* in enriched food cultures was compared for the first time in this study. The intensity of the PCR amplicons generated by the four PCR primer sets varied across the nine stomacher bags of enriched cultures originating from the three food types. Since the primer sets described by Ito et al^13^^)^, Hidaka et al^18^^)^, and Müller et al^15^^)^, yielded PCR amplicons with varying intensities, including faint bands, the result interpretation as PCR-positive or PCR-negative could be challenging. Conversely, the PCR primer described by Yamamoto & Echeverria^3^^)^ consistently yielded a PCR amplicon intensity comparable to that of the positive control or a PCR-negative result, suggesting that the results obtained from this PCR primer set were easy to interpret. These discrepancies could potentially originate from the *astA* gene sequence diversity, as several variants have been previously reported^2^^,^^9^^,^^23^^,^^24^^,^^25^^,^^26^^)^. However, the *astA* variant associated with foodborne outbreaks remain to be elucidated. Moreover, it is also possible that the sample exhibiting faint or weak bands contained a low abundance of *E. coli* strains harboring *astA* in the enriched culture. Consequently, isolating *E. coli* strains harboring *astA* from such cultures could be challenging due to the limited number of target bacterial cells. To assess the performance of different PCR enzymes, the primer set described by Yamamoto & Echeverria^3^^)^ was used. Enriched cultures from stomacher bag Nos. 1 and 3 of okra No. 4 were selected for this evaluation, since these enriched cultures had previously shown no *astA*-specific PCR amplicons with the primer set described by Yamamoto & Echeverria^3^^)^ (**Table 4**), and all the colonies grown on the agar plates were presumed to be PCR-negative. Among the tested enzymes, Quick Taq HS DyeMix yielded an *astA*-specific amplicon to the positive control and did not yield any non-specific amplicons in the PCR products from food cultures, whereas the other enzymes yielded weaker amplicon intensities to positive control or generated non-specific bands. Furthermore, Quick Taq HS DyeMix includes a loading buffer, facilitating the electrophoresis step compared to the other enzymes. Since the TaKaRa Ex Taq was used for the initial evaluation of the PCR primer sets, the three primer sets were subsequently re-evaluated using Quick Taq HS DyeMix to ensure consistency.

False positive results in PCR assays are often suspected when PCR detection is positive, yet bacterial isolation is negative. Furthermore, as discussed above, isolating the target bacteria from enriched cultures that exhibited faint or weak amplicons in the PCR assay proved challenging. Accordingly, in evaluating the PCR primer sets using Quick Taq HS DyeMix, the correlation between isolation and PCR results was examined. The group 1 samples, where *E. coli* strains harboring *astA* were not isolated, tested PCR-negative with the primer set described by Yamamoto & Echeverria^3^^)^; however, they yielded positive or weakly positive results with the other primer sets. Notably, in these two samples, the primer set described by Yamamoto & Echeverria^3^^)^ was the only one to show consistent results between isolation and PCR, although the other six samples were PCR-positive and isolation-negative. For both PCR-positive and PCR-negative samples, developing an isolation method specifically targeting *E. coli* strains harboring *astA* would be advantageous for effectively isolating these strains from enriched cultures.

Since the combination of the primer set described by Yamamoto & Echeverria^3^^)^ and the Quick Taq HS DyeMix DNA polymerase demonstrated efficiency for detecting *astA* in enriched food cultures, the specificity and sensitivity of this assay were further investigated. This PCR assay exhibited high specificity, and its detection limit (≤5 log CFU/mL) is comparable to or even superior to conventional PCR methods. Furthermore, no PCR inhibition by the specific food matrices tested was observed in this study.

In conclusion, the PCR assay employing the primer set described by Yamamoto & Echeverria^3^^)^ and Quick Taq HS DyeMix is a valuable tool for detecting *E. coli* strains harboring *astA* in food samples. The amplicon generated by this PCR assay and visualized after gel electrophoresis is specific for detecting *astA*. Since this PCR assay exhibited high sensitivity and specificity for *astA* detection, its application is anticipated to enhance the detection and subsequent isolation of *E. coli* strains harboring *astA* in foodborne infection outbreaks.

## Supplementary materials
