# Supplementary material for: An Optimized PCR Assay to Detect Escherichia Coli Harboring the astA Gene Encoding the Enteroaggregative E. coli Heat-Stable Enterotoxin 1 in Various Food Matrices
Source: Food Saf (Tokyo). 2025 Dec 19;13(4):70–7. doi: 10.14252/foodsafetyfscj.D-25-00013 (PMC12718105; doi:10.14252/foodsafetyfscj.D-25-00013)
Supplement: Supplementary file 1 [file foodsafetyfscj-13-4-70-s001.pdf]

Supplementary materials

Table S1. Bacterial strains used in this study and the result of PCR assay*

Species	Number of strains	Number of PCR-positive
Arcobacter butzleri	1	0
Arcobacter cryaerophilus	1	0
Arcobacter skirrowii	1	0
Bacillus cereus	1	0
Campylobacter coli	1	0
Campylobacter jejuni	1	0
Citrobacter freundii	1	0
Enterobacter cloacae	1	0
Enterobacter aerogenes	1	0
Escherichia albertii	1	0
Escherichia coli		
Type strain	1	0
E. coli strain harboring *astA*	9	9
Escherichia fergusonii	1	0
Escherichia hermannii	1	0
Hafnia alvei	1	0
Klebsiella oxytoca	1	0
Morganella morganii subsp. *morganii*	1	0
Proteus mirabilis	1	0
Salmonella enterica subsp. *enterica* serovar Typhimurium	1	0
Shigella boydii	4	0
Shigella dysenteriae	2	0
Shigella flexneri	1	0
Shigella sonnei	1	0
Staphylococcus aureus	1	0
Vibrio parahaemolyticus	1	0
Yersinia enterocolitica	1	0
Yersinia pseudotuberculosis	1	0
Total	39	9

* PCR assay by Yamamoto & Echeverria was performed with Quick Taq HS DyeMix.
